# Architectural and Technological Improvements to Integrated Bioprocess Models towards Real-Time Applications

**DOI:** 10.3390/bioengineering9100534

**Published:** 2022-10-09

**Authors:** Christopher Taylor, Barbara Pretzner, Thomas Zahel, Christoph Herwig

**Affiliations:** 1 Körber Pharma Austria GmbH, 1070 Vienna, Austria; 2 Research Area Biochemical Engineering, Vienna University of Technology, Gumpendorferstrasse 1a, 1060 Vienna, Austria; 3 Competence Center CHASE GmbH, Altenbergerstraße 69, 4040 Linz, Austria

**Keywords:** integrated process model, digital twin, Pharma 4.0, bioprocess, control strategy, upstream, downstream, real time, holistic model, data science

## Abstract

Integrated or holistic process models may serve as the engine of a digital asset in a multistep-process digital twin. Concatenated individual-unit operation models are effective at propagating errors over an entire process, but are nonetheless limited in certain aspects of recent applications that prevent their deployment as a plausible digital asset, particularly regarding bioprocess development requirements. Sequential critical quality attribute tests along the process chain that form output–input (i.e., pool-to-load) relationships, are impacted by nonaligned design spaces at different scales and by simulation distribution challenges. Limited development experiments also inhibit the exploration of the overall design space, particularly regarding the propagation of extreme noncontrolled parameter values. In this contribution, bioprocess requirements are used as the framework to improve integrated process models by introducing a simplified data model for multiunit operation processes, increasing statistical robustness, adding a new simulation flow for scale-dependent variables, and describing a novel algorithm for extrapolation in a data-driven environment. Lastly, architectural and procedural requirements for a deployed digital twin are described, and a real-time workflow is proposed, thus providing a final framework for a digital asset in bioprocessing along the full product life cycle.

## 1. Introduction

### 1.1. Background of Integrated Process Models

In recent years, bioprocess research & development has been seeking to speed up the time to market through the advanced analytical modeling of development data. Of particular focus is the ability to predict final drug quality with minimal data input. One promising technology is integrated process models (IPMs, also referred to as holistic models). These are in silico model frameworks of multistep processes used to perform simulations that predict the behavior and outcome of a full process chain [1,2]. A digital twin (DT) is effectively an extension of this technology, which feeds the resulting output data back into the model in real time [3]. The key components in building a DT are the physical asset (i.e., the process), the digital asset (DA, i.e., the model), and the bidirectional connectivity between them to exchange data and enable a control loop [4]. This concept was mentioned as early as 2003, but has been receiving increasing attention in industry in recent years, not least in the pharmaceutical and biotechnology sectors [1,5,6,7,8,9]; extensive descriptions can be read elsewhere [10]. With IPMs serving as the DA component to a DT, the industrial potential is clear. By leveraging a digital copy of the process where simulations replace physical experiments limited only by computational power, process success can be maximized, and failures may be swiftly mitigated. For bioprocesses and bioproduct lifecycles, an IPM can substantially shorten development and improve quality both in terms of speed to market and manufacturing success rates [1,11,12].

Modeling individual process steps or unit operations (UOs) in succession have a long history, starting from simple linkage studies [13,14,15]. However, until recently, few comprehensive frameworks had been established in biopharma development. In 2017, a baseline IPM technology was proposed (IPM 1.0) to serve as a bioprocess ‘life cycle companion’ with the potential to be a DA. In this framework, the bioprocess is constructed by concatenating individual UO models in a central repository that statistically depicts the entire process in the correct process order [16]. Each model represents a single UO with process parameters (PPs) as input factors and critical quality attributes (CQAs) as responses. Once established, the model serves as a “mirror” to the physical asset [10]. Monte Carlo (MC) applications are then leveraged to simulate the propagation of error across the process on the basis of the variation in input factors and subsequent responses. The final simulation result is obtained at drug substance. 

IPM 1.0 was trained primarily on specific clearance measurements in characterization data at a small scale – usually performed within a Design of Experiment (DoE) – and in the limited available large-scale (LS) manufacturing data, though the framework also accepted mechanistic and hybrid models. The two scales were fitted into separate matrices and combined to create a single output prediction per variable. This two-matrix system has the disadvantage that the two models require a secondary mathematical step to combine the results. This both leaves any scale offset unaddressed and results in a non-normally distributed result during simulation due to the multiplication and division of the random variables. Equation (1) defines the *j-th* CQA’s predicted specific clearance $(\hat{SC)}$ as a ratio of the *SLC_l_* (large-scale) and mean *SLC_DoE_* (small-scale) results, at a given process parameter setting ($\hat{SC}\left(PP_{i}\right)$).

(1) $${\hat{SC}}_{j}=\hat{SC}\left(PP_{i}\right)\times \frac{\hat{SC}\left(SLC_{l}\right)}{\hat{SC}\left(\bar{SLC_{DoE}}\right)}$$

In the population of simulated values where these terms are both normal distributions, the resulting simulated *SC_j_* distribution is Cauchy distribution. Furthermore, this ratio is multiplied by the predicted PPs specific clearance ($\hat{SC}\left(PP_{i}\right))$. The final distribution is, therefore, a product distribution that is proportional to, but not per se, a normal distribution. This relationship can potentially give a less precise estimator of the final resulting simulated distributions and be biased versus a normally distributed predicted result [17].

IPM 1.0 also addressed only non-scale-dependent variables, such as those representing specific clearances, as mentioned above. This is useful in establishing the technology, as all the responses are easily linked with identical units across all UOs. This method also circumvents the issue of modeling volumes that are usually difficult to model since they are controlled by manufacturing and organization considerations. As a consequence, however, this limits the modeling of key process attributes such as *Yield* or *Product Amount,* which is of particular business interest. 

### 1.2. State of the Art for Holistic Bioprocess Models

Since the introduction of IPM 1.0, additional MC applications have been introduced that target specific regulatory deliverables. These include estimating out-of-specification (OOS) results, defining control strategy elements such as proven acceptable ranges (PARs), and linking sensitivity analyses to quality-by-design (QbD) milestones [11].

Recent alternative approaches have also been studied with the goal of both comprehensively describing the process chain and meeting regulatory submission requirements. 

Flowsheet models have been proposed for small-molecule pharmaceuticals that, while very similar to the IPM 1.0, differ in the selection of linkage variables used to concatenate the UOs. In one recent case study, using models based on first principles, output responses were directly translated into input variables for the subsequent UO’s mechanistic model [18]. This approach has the flexibility that response variables do not necessarily need to be simulated across all UOs. Indeed, certain responses may be modeled only for use as an input factor in a different response’s model, with all pathways leading to a potentially different final output CQA. This permits modeling flexibility, where each response is not necessarily assayed across all UOs. In bioprocess applications, it is of particular importance to consider a mechanism for estimating parameter and model uncertainty in the prediction, as heightened variation is inherent to the biosystem and significantly impacts the precision of the predictions [19]. 

Toolboxes of hybrid modeling techniques have recently been proposed that allow for maximally parsing relevant values at different scales. In one instance, an upstream UO was assessed at four different scales, with a sequential procedure for analyzing the multidimensional data to proceed with each subsequent experiment in a pathway optimized to reduce experimental load. This harbors the advantage of directly addressing the quantitative and qualitative differences of scale, and works towards a holistic process evaluation. It is efficient to combine the scales (as opposed to modeling them separately) for three reasons: the degrees of freedom increase, the manufacturing design space is more accurately represented, and the scale offsets can be measured directly. Nonetheless, the framework still needs to offer a linkage between the different UOs in order to address the ultimate impact on CQAs at drug substance [20].

This linkage has recently been assessed in a Bayesian framework for concatenating UOs. Here, the outcomes of potentially multiple models (or one model trained on bootstrapped data) are leveraged as uninformed prior distributions and, using Markov chain MC algorithms, are transformed into a posterior distribution. Random sampling from this distribution is used for the transfer to subsequent UOs. One advantage here is the combination of multiple models per UO, which may be useful in creating more robust predictive outcomes, especially in data-poor environments [21]. One consideration to add to this framework is the prediction of extreme model outputs. Such values are likely outside the training dataset range, but are probabilistically inevitable. This is particularly important for the variable with the most impact on the linkage between UO models. In case of an extreme linkage value, a de facto extrapolation occurs in the second UO, which is highly discouraged in data-driven environments. In any future manufacturing state, potentially extreme results and their impact on subsequent UOs should be considered on risk management grounds. This extrapolation is not performed at the moment in any data-driven holistic process model of which we are aware.

Lastly, to the best of our knowledge, none of these recent bioprocess use cases proposes an integrated real-time application, particularly in commercial manufacturing where the effects of PP deviations in an ongoing process can be simulated onto final drug substance specifications. Such a prediction would provide actionable information to optimize or mitigate process outcomes. Enabling this application would have the potential to increase the process success rate and shorten the time to market. The collection of these innovations would provide a robust platform on which to build a real-time simulation, prediction, and feedback loop. Such a technology would ultimately provide bioprocesses with a plausible DT.

### 1.3. Suggested Improvements

Each of the recent approaches has significant advantages within the context of bioprocess development requirements. This contribution aims to leverage them collectively to establish a novel IPM that solves numerous challenges in one framework:

- Simplification and improvement of the IPM 1.0 two-matrix procedure.
- Combination of manufacturing- and development-scale data.
- Establishment of scale-dependent variable procedure.
- Improvement of model uncertainty intervals.
- Creation of an extrapolation procedure for non-controllable parameters.
- Description of a real-time DA application.

This contribution proposes building the above improvements on the conceptual backbone of IPM 1.0. The resulting technology would lead to a plausible DA for a bioprocess development DT. Computational comparison with previous approaches is not within scope here, as the primary goal is to create a framework that combines all the above improvements.

**Figure 1:**
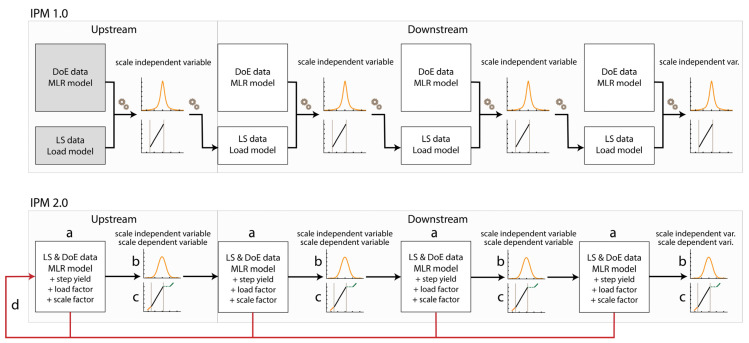
(top) Original IPM technological process flow (IPM 1.0). (bottom) Proposed collection of IPM innovations (IPM 2.0). IPM 2.0 differs from IPM 1.0 in the following improved areas: (**a**) robust and simplified data model, (**b**) addition of scale-dependent responses, (**c**) conservative extrapolation procedure for multiple linear regression (MLR) models, and (**d**) real-time feedback loop (depicted as a red line).

Figure 1 compares the above-discussed limitations with the proposed innovations. The top model shows the structure of the IPM 1.0, whereas the lower model depicts the proposed innovations (IPM 2.0) to be discussed in this collaboration.

## 2. Materials and Methods

### 2.1. Software

The IPM was developed with commercially available software PAS-X Savvy 2022.01 (Körber Pharma Austria GmbH, Vienna, Austria). This software uses Python 3.79 as a base (Python Software Foundation, available online: https://www.python.org/, accessed on 20 January 2022). The procedures below were built onto the framework of the IPM 1.0.

### 2.2. Data

A case study was prepared with an industry partner to assess the proposed procedures as a proof of concept. A recombinant protein production process in a mammalian cell culture was provided that had been developed and characterized with a limited number of at-scale manufacturing runs. The model contains one primary upstream UO and seven downstream UOs, followed by final results at drug substance.

The downstream process consists of the following UOs: a chemostat bioreactor (UO1), followed by a filtration step (UO2), a concentration step (UO3), a virus inactivation step (UO4), a capture chromatography step (UO 5), filtration (UO6), and two polishing chromatography steps (UO7 and UO8). 

The primary response for the case study is *Step Yield*, as it best leverages and displays the proposed innovations, further discussed in the Results section. 

The available statistical models for each UO are summarized in Table 1 and are characterized in more detail in Table S1. PP is a model built upon process parameters, but not including an input load value. A *Step Yield* model is only missing from UO4, as no statistically significant model was found. The raw data for *Step Yield* for each UO are described in Table S2.

### 2.3. IPM Data Model

This collaboration builds on the IPM 1.0 technology, adapting the general concept of combining a lab-scale model with manufacturing process data. The lab scale provides the bulk of the investigated design space, and the manufacturing data provides the primary UO linkage. IPM 1.0 proposes a two-matrix system based on scale-independent variables (specific clearances, SC, downstream).

The two required data matrices are the following: a standard p×n matrix (where *p* is the number of parameters and *n* is the number of runs) of small-scale DoE data that explore the investigated design space. Many DoEs, in our experience, are modeled in the individual UO and have no connection to the previous UO. The large-scale data matrix is a 1×n matrix with the only factor being the incoming specific load of a given CQA to be regressed against the output CQA. This depicts a de facto transfer function between UOs [2]. 

Equation (2) defines the specific load clearance model (*SLC*) of the *j-th* CQA as the *i-th* UO’s pool values in percentage (%) divided with the *i-th* UO’s load density (which itself is load divided by column volume CV).

(2) $$SLC_{j}=\frac{\left(\frac{CQA_{j,i\;load}}{\mathrm{CV}}\right)}{CQA_{j,i\;pool}}\;$$

The combination of the two models occurs only during the simulation phase and proceeds according to Equation (1). 

## 3. Results

### 3.1. Data Model

A simpler and more robust data model can be established, given the availability of certain additional information about the scale and starting material. All scale data can be combined in a single matrix and subsequently fitted by a single model provided that two new columns are also added: *Scale* and *CQA_load_*.

*Scale* is treated as a fixed categorical factor, thereby having the benefit of capturing any scale offsets within the model. In addition to providing this important scale comparison as a simple regression coefficient, the *Scale | Large* level can be selected as the prediction setting during the MC procedure, thus always simulating under manufacturing-scale conditions.

**Figure 2:**
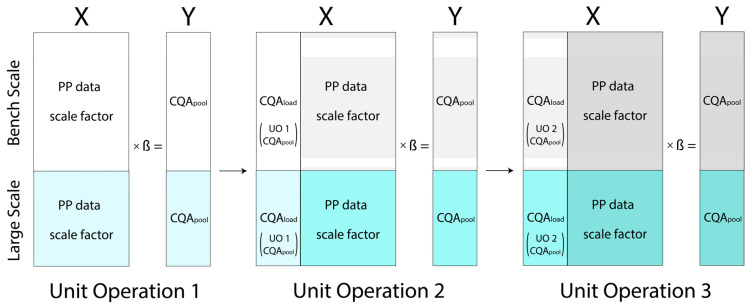
Proposed data model for the IPM. Small-scale DoE data (greyscale boxes, top) and large-scale manufacturing data (colored boxes, bottom) are in the same matrix with an added categorical scale factor. Each modeled CQA has a unique additional factor called *CQA_load_*, which is the *CQA_pool_* value from the previous UO.

*CQA_load_* refers to the pool value of the CQA from its precursor UO. That is, the starting material value for any given CQA is used to model its impact on the pool CQA (*CQA_pool_*) in the current UO. This factor does not refer to *Load Concentration* (i.e., the desired molecule amount over volume) or *Load* *Density* (i.e., the desired molecule over resin volume/filter area) necessarily, but rather each CQA’s own starting material. The upshot is the creation of an individual factor matrix X for each CQA, as seen sorted by color in Figure 2. 

The regression is now described in Equation (3): the predicted CQA (${\hat{y}}_{i})$ is generated by model intercept $\beta_{0}$ plus all investigated factors (x_i_) and their respective coefficients $\left(\beta_{i}\right)$ plus the error term (ε). The two non-PP terms (*CQA_load_* and *Scale*) provide the linkage of the model to both manufacturing scale and the subsequent UO. Architecturally, the process consists of statistical model objects representing the UO models. This permits a simple modular build-up of the full model and replacement upon refitting with new data.

(3) $${\hat{y}}_{i}=\beta_{0}+\beta_{load}x_{load}+\beta_{scale}x_{scale\;}+\beta_{1}x_{1\;}+\ldots \;\beta_{n}x_{n\;}+\varepsilon$$

When performing MC simulations, *CQA_load_* serves as the mathematical link between the precursor and current UOs. If the *CQA_load_* is nonsignificant in the regression model, there is no mathematical link between the UOs for that CQA. 

### 3.2. Extrapolation Procedure

In the above case, there is likely, nonetheless, a point at which the relationship is indeed quantifiable even if it is outside the investigated design space. Guarding against overlooking such a relationship requires extrapolation. As discussed, for data-driven models, extrapolation is discouraged in the absence of established first principles or process knowledge, since data alone is agnostic to behavior outside the observed data [22]. DoEs purposefully vary PPs outside typically observed manufacturing ranges. However, this space is limited by resources and knowledge. Additionally, not all PP can be specifically controlled, such as the *CQA_load_*, which contains propagated variation from all previous UOs. It is generally assumed that *CQA_load_* has a quantifiable influence on the CQA value in the following UO (*CQA_pool_*), even if not detectable in the design space. Without a mechanism to account for this uncertainty, the DA can only predict within already observed data. 

**Figure 3:**
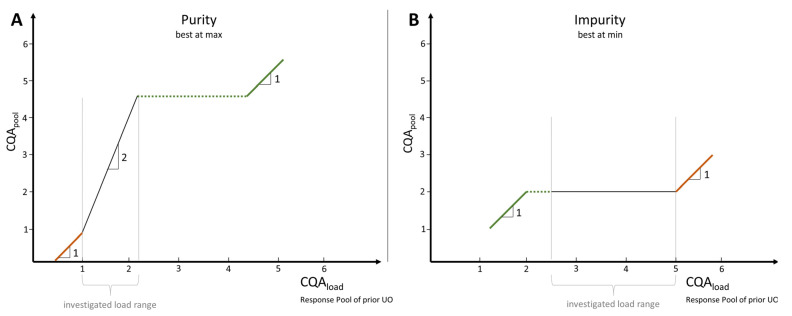
Visualization of *CQA_pool_* value correction strategy for (**A**) purities and (**B**) impurities. (**A**) If the simulated *CQA_load_* value is beyond the investigated load range (grey area), but below the maximal observed value of *CQA_pool_*, then *CQA_load_* is purified up to the maximal *CQA_pool_* value at most, as depicted by the green dashed line. If, on the other hand, the simulated *CQA_load_* value already exceeds the maximal observed *CQA_pool_* value, no further purification takes place, and the *CQA_load_* value equals the *CQA_pool_* value, indicated by the green solid line. Conversely, if the simulated *CQA_load_* value is below the investigated load range, then no purification takes place, and *CQA_load_* corresponds to the *CQA_pool_* value, visualized by the orange solid line. (**B**) The correction of the impurity *CQA_pool_* values follows the same strategy as for the purities, only exactly reversed.

Naive extrapolation of a data-driven model is indeed associated with extreme statistical uncertainty [23], but extrapolation may be constrained by conservative process-based assumptions that allow for a reasonable worst-case assessment of the quantified relationships [24]. Specifically for bioprocesses, this constraint must be at least severe enough to satisfy risk management in bioprocess development. Therefore, a linear stepwise extrapolation strategy for the simulation of *CQA_load_* values is proposed here. This strategy differs depending on whether the CQA is categorized as impurity or purity and whether the simulated value is below or above observed measured values as depicted in Figure 3.

#### 3.2.1. Purities (Best at Max) 

##### Above the Observed Load Range

If the simulated *CQA_load_* value ($\hat{load_{i}}$) is above the observed load range (max(load)) but below the observed maximal *CQA_pool_* value ($y_{max}$), the resulting simulated *CQA_pool_* value (${\hat{y}}_{i}$) is corrected by the *CQA_load_* value coefficient ($\beta_{load}$) multiplied by the offset between the maximal observed load range and the simulated *CQA_load_* value, as shown in Equation (4), depicted as the green dashed line in Figure 3A. This is a conservative assumption that ensures that no further purification occurs when *CQA_load_* values are purer than those in the pool. Thus, *CQA_pool_* values are constrained to the maximal observed *CQA_pool_* value.

(4) $${\hat{y}}_{i,corrected}={\hat{y}}_{i}+\beta_{load}*\left(\max\left(load\right)-\hat{load_{i}}\right)$$

If the simulated load value exceeds the observed *CQA_pool_* value, the excess load is added to the corrected *CQA_pool_* value (${\hat{y}}_{i,corrected}$), as described in Equation (5). That is, the *CQA_load_* value is simply passed through to the pool and no further clearance takes place, depicted as the green solid line in Figure 3A. It was assumed that the purity no longer decreased, and that a 1:1 propagation occurred.

(5) $${\hat{y}}_{i,corrected}={\hat{y}}_{i,corrected}+\left|\hat{load_{i}}\right|-\left|y_{max}\right|$$

##### Below the Observed Load Range

If the simulated *CQA_load_* value is below the investigated load range, no purification takes place, as visualized by the orange solid line in Figure 3A. This conservative correction, as described in Equation (6), results in the *CQA_load_* value not being purified, and the same concentration arriving in the pool.

(6) $${\hat{y}}_{i,corrected}={\hat{y}}_{i}+\left(1-\beta_{load}\right)*\hat{(load_{i}}-\min\left(load\right))\;$$

#### 3.2.2. Impurities (Best at Min)

##### Above the Observed Load Range

For impurities, the conservative approach follows that no clearance occurs if the simulated *CQA_load_* values are above the investigated load ranges, as described by Equation (7) and depicted by the orange solid line in Figure 3B.

(7) $${\hat{y}}_{i,corrected}={\hat{y}}_{i}+\left(1-\beta_{load}\right)*\hat{(load_{i}}-\max\left(load\right))$$

##### Below the Observed Load Range

If the simulated *CQA_load_* is below the investigated load range, but not below the observed minimal *CQA_pool_* value, the simulated *CQA_pool_* value is forced to the minimal observed *CQA_pool_* value, as visualized as the green dashed line in Figure 3B.

(8) $${\hat{y}}_{i,corrected}={\hat{y}}_{i}+\beta_{load}*\left(\min\left(load\right)-\hat{load_{i}}\right)$$

If the simulated *CQA_load_* value is below the minimal observed *CQA_pool_* value, the *CQA_load_* value is passed to the pool without any clearance, as described and depicted as a green solid line in Figure 3B.

(9) $${\hat{y}}_{i,corrected}={\hat{y}}_{i,corrected}+\left|\hat{load_{i}}\right|-\left|y_{max}\right|$$

### 3.3. Uncertainty Intervals

Where implemented, process models (including the IPM 1.0) tend to estimate uncertainty by sampling the confidence interval of the individual models. These intervals determine the uncertainty of the model mean, but are not optimized for predicting manufacturing data over many batches. Therefore, tolerance intervals were added as the default prediction setting for the IPM 2.0 data model on the basis of an established fixed-effect regression model implementation [25]. As such, both the confidence and the future coverage of the prediction are considered in the total variation, which, to the best of our knowledge, is not currently used in any equivalent integrated process model.

### 3.4. Scale-Dependent Variable Simulation Procedure

IPM 1.0 did not describe the modeling and simulation of responses other than specific clearances, which have scale-independent units that do not change over the UOs. To test the feasibility of an alternative pathway for nonspecific or scale-dependent variables, we propose modeling the product amount at the end of the upstream process (i.e., Harvest) and then adjusting via the individual UOs to simulate *Step Yields* without requiring the separate modeling of volumetric changes. This entails partially removing the response from the process model chain while still retaining the impact by process parameters. *CQA_load_* is replaced by a variable that was only modified by the model output and is assayed through as much of the process as possible; in this case, *Global Yield*. The procedure is below and is generalizable to any variable that has a component (i.e., *Volume*) not described in the process models themselves.

**Figure 4:**
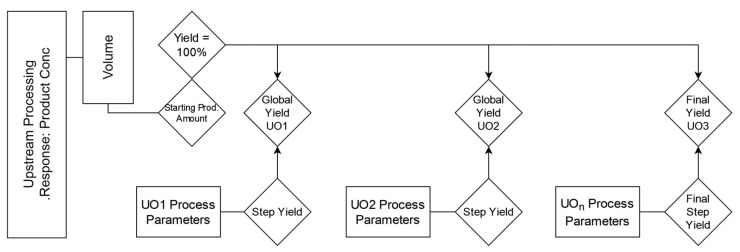
*Step* and *Global Yield* procedure as a model for scale-dependent variables. *Product Amount* is determined from upstream processing and considered to be 100%. All subsequent *Step Yields* may be removed from the linkage of UOs. *Step Yields* modify *Product Amount* by percentage recovery, which in turn modifies the *Global Yield*, which is updated after each UO towards a final *Global Yield* metric at DS.

As seen in Figure 4, the yield may be seen as a combination of *Step Yield* and *Global Yield*. The proposed procedure during the IPM MC simulations is as follows:

1. *Concentration* at harvest converted *Product Amount* to amount either by a known fixed volume or by sampling a distribution of feasible volumes.
2. *Product Amount* becomes the first downstream UO pool value.
3. *Step Yields* are fitted in the individual UO data, unconnected to the precursor UO, as per Equation (10).
4. *Step Yield* is multiplied by the current *Product Amount,* and a new *Product Amount* is calculated.
5. The new *Product Amount* remains outside the model loop and is adjusted by the subsequent UO *Step Yield* predictions.
6. In addition to modifying *Product Amount*, a new attribute is produced: *Global Yield*, which is the current UO’s *Product Amount* divided by the original harvest *Product Amount* (Equation (11)).
7. The above process repeats until drug substance and a final *Global Yield* is produced, defined as the ratio of the final *Product Amount* to the original (max) *Product Amount*.

(10) $$Step\;Yield_{i}=\frac{Amount_{i}}{Amount_{i-1}}$$

(11) $$Global\;Yield_{i}=\frac{Amount_{i}}{Amount_{0}}$$

Defining the acceptance criteria for *Global Yield* allows for the establishment of intermediate acceptance criteria for *Step Yields* via parameter sensitivity analysis. While this result does not produce a final *Product Concentration* per se, the final *Product Amount* may be modified by final volume adjustments as needed to arrive at a concentration.

### 3.5. Feasibility Case Study Results

A proof of concept was performed using the dataset shared by an industry partner described in the Methods section. This case study evaluates *Step Yield* to show the feasibility of the above-mentioned improvements. The *Step Yield* IPM was built successfully, containing all UO models. For each UO in the IPM chain, a *Step Yield_load_* (i.e., the *Step Yield* from the precursor UO) design space was determined and divided into equidistant points called grids. The grid size covers a proposed range of likely *Step Yields* from the precursor UO, purposefully chosen to be outside the observed *Step Yield* ranges. The holistic process was then simulated at each grid per UO. With each simulation, the process was allowed to culminate at DS, and the final result was compared to a *Global Yield* OOS limit determined by a process expert. After repeating the simulation 200 times per grid size, a final %OOS value was obtained.

**Figure 5:**
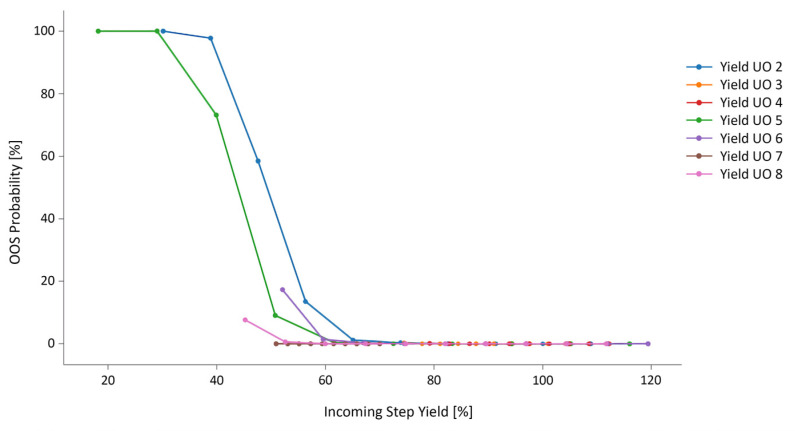
Parameter sensitivity analysis for *Step Yield* per UO. The y axis shows the proportion of OOS results based on the global yield drug substance specification, which is based on simulated incoming step yields per UO. Step yield results were extrapolated 10−20% outside the currently observed results to test the feasibility of the extrapolation procedure.

**Figure 6:**
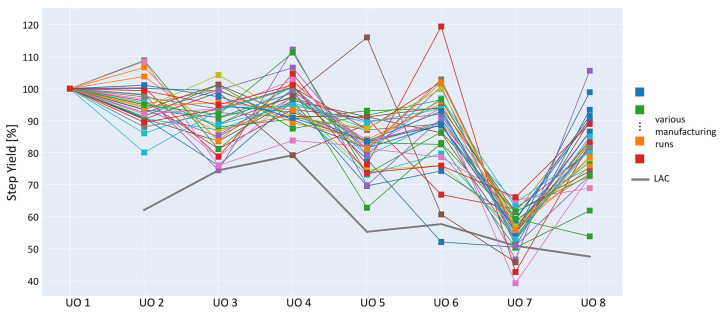
Parallel coordinate plot with results of parameter sensitivity analysis for the establishment of intermediate acceptance criteria for the *Step Yield*. Available manufacturing data are shown in various colors sampled from several campaigns/scales. The proposed intermediate acceptance criteria are marked in dark gray. Acceptance criteria were automatically generated across all intermediate UOs via the likelihood of meeting DS specifications predetermined by process experts.

The results are shown in Figure 5 and Figure 6. In Figure 5, the simulated *Step Yields* and their respective OOS results (%) are shown, which include extrapolated *Step Yields* (no OOS was observed in the data). For most UOs, there exists an incoming *Step Yield* at which the OOS rate starts to steeply rise, i.e., the *Global Yield* specification is no longer attainable. Process experts were then able to fix the *Step Yield* acceptance criteria to the point at which the OOS increase passes 5%. 

In Figure 6, all observed data are plotted against the results of the above IPM-derived intermediate acceptance criteria. The acceptance criteria show a risk of increased OOS in the penultimate UO, which the process experts investigated and confirmed as a limitation of the current process. Subsequent actions were taken to adjust the process parameters to meet this new limit. The results were confirmed by process experts to be used in support of the final intermediate acceptance limit establishment.

There are instances of *Step Yields* with results >100%. Discussions were held with the subject-matter experts, and these artifacts stemmed from variation within the analytical method, i.e., variation in the load and pool values where both results were near 100%. The plot may also indicate high fluctuations of *Step Yields* between UOs. However, these results should be interpreted as independent of the precursor *Step Yields*. Since *Step Yields* always have a starting load amount of 100%, it is not unexpected to have large differences in mean yield in different UOs. 

Thus, leveraging a *Global Yield* DS specification and all the improvements described above, plausible practical results were generated leading to adequate *Step Yield* intermediate acceptance criteria. 

## 4. Discussion 

### 4.1. Data Model 

The simplification from the original two-matrix procedure into a single matrix aims to better meet the bioprocess development need of extracting data from differing scales. *Scale* is assayable directly in the same model where design space and UO linkage are fit. The manufacturing-based univariate model is replaced with a multivariate model, reducing the overall error term, since varying PPs are controlled.

The single matrix of course also reduces effort in compiling the data for a DA. Other than the addition of the individual *CQA_load_*, the matrix requires only the necessary preprocessing for standard DoE-based regression analyses [26]. Moreover, it represents an improvement on classic linkage studies where multiple UOs must be modeled as one unit. Here, UOs may be modeled fully separately with no matrix overlap [27] while maintaining the CQA linkage. This simplicity also protects against data entry errors between the scales. Lastly, the data model provides normally distributed results since there is no longer a potential for product or Cauchy distributions due to the manipulation of the two models. 

Newly arising higher-order terms may also be of interest, such as the interaction between *Scale* and PPs, which would give insight into the behavioral changes between scales rather than a simple offset. This could be used to significantly strengthen the conclusion of scale-down model qualifications, which are normally univariate. However, additional degrees of freedom are required for these terms, and, given the generally minor range of process parameter variables at a set point, the likelihood of an unfavorable correlation structure or even a singular matrix increases. Cost–benefit analysis should be undertaken before adding further terms. 

There are further limitations to the current procedure that must be carefully considered. The two additional factors that were added to the matrix (i.e., *Scale* and *CQA_load_*) are often not explored factors in original DoEs. Specifically, this information is often available, but was not included in the original design. The reassessment of appropriate design metrics (i.e., correlation, aliasing, power) is, therefore, required to ensure that the regression may still be performed. Less often, *CQA_load_* is not tested at all. In this case, it is not possible for the data model to populate without additional context. Therefore, it is strongly advisable to include these factors a priori in statistically underpinned designs or minimally assess the data environment before beginning to fit models. 

### 4.2. Extrapolation Procedure

The extrapolation procedure is a useful tool in bioprocess characterization since it allows for decision making within a risk management framework, even in the absence of data. The worst case defined in this procedure can allow for useful inferences about the edges of the system. Practically, it allows for conservative intermediate acceptance criteria and parameter limits to be provisionally established; these limits must otherwise be constrained within the current UO’s observed data range.

Furthermore, this extrapolation procedure can be used as a stress test for subsequent UOs. Upon generating an extreme value, all subsequent UOs may process much more extreme input variables than those in their observed training data. Some of these UOs were physically designed to manage these unexpectedly high values and thus produce models that can easily purify excess material. Thus, one of two outcomes may be observed. Unexplored edges of the system show weaknesses in downstream steps. If worst-case results are easily managed in subsequent UOs, further experimental effort may be reduced as the risk of OOS is lessened.

The primary limitation is that the physical behavior of the process under extreme values is not known, and the system may react differently to the extrapolation assumptions. While this procedure utilizes worst-case assumptions, thereby leaning on patient safety, these strict assumptions may nonetheless not hold upon fitting new data. 

### 4.3. Scale-Dependent Variables

Simulating scale-dependent variables holistically over the process expands the application of the IPM to variables describing product quantity or process performance. The upshot is side stepping complexities arising in those variables having a volume component (or any component) that is controlled in a way that is not simple to define in a procedure or algorithm.

One operational disadvantage, in our experience, is that a lack of procedural strictness (such as in the case of a CQA dilution or concentration) is occasionally leveraged by operators towards increased manufacturing flexibility or buffer in achieving scale-independent results. In certain cases, this flexibility is preferred in operations; thus, the buy-in to this procedure may be dependent on the management’s view of quality or yield outcome favorability. 

### 4.4. Digital Environment and Real-Time Applications

Each complex step in bioprocess manufacturing potentially impacts the quality of the final product, yet state-of-the-art practices focus on the static outputs of individual UOs rather than on a holistic process model, particularly with regard to potential real-time applications [1,4,28,29,30,31]. Having so far discussed the innovative improvements to the IPM technology, it is now important to better define the framework for real-time DA deployment.

As previously discussed, by simplifying the data format, individual UO models can now easily be refitted by updating the single data matrix; thus, new predictions can be seamlessly conducted. With the physical process holistically depicted in silico and with a simple procedure to update the models, there needs only to be a framework for the feedback loop in real time.

**Figure 7:**
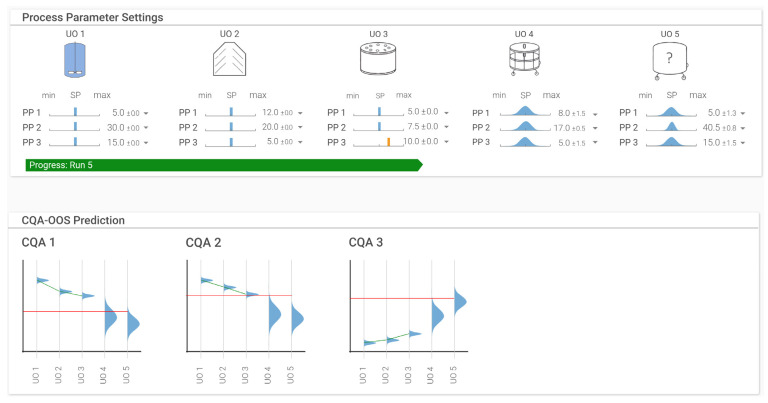
Proposed control panel for IPM use in a real-time environment. Process parameters may be controlled manually or through targeted APIs to either create a prediction around the process parameter (set point plus expected normal operating variation) or to bring in the discrete value when the PP setting is known. Predictions via MC simulations around the chance of specification conformity can be updated immediately. Refitting the models may also be performed in real time or at regular intervals.

Figure 7 shows a proposed graphical user interface for an IPM depicting the UOs in the upper half of the plot and the resulting predictions of the CQAs across the UOs in the lower half. A real-time workflow should proceed as follows: 

The process begins at UO1 and ends at UO5, as shown in the upper half of Figure 7. At the start of the process, when no UO has been executed, the prediction of the resulting CQAs is based on sampling a most likely setting (i.e., normal distribution around the set point) of the PPs for each UO based on the variation of the large scale training dataset. This PP uncertainty maximally propagates through the prediction of the resulting CQAs. As the process progresses, however, and the actual PP settings are fed into the IPM (either manually or by automated import using API interfaces), these PP values become fixed points rather than distributions. Subsequent CQA predictions naturally become more accurate. By the last UO, the accuracy of the predictions should equal the accuracy of the individual UO model. 

Figure 7 is, therefore, a snapshot of the process at a given time. The process is currently at UO3, and the uncertainty of the PP from UO1 to UO3 was set to 0, as the PP values are already known. These settings are immediately used to repredict the CQAs, creating a feedback loop and allowing for a reaction to the new conditions. If, for example, a PP is performed outside the normal operating ranges (shown as the orange bar at UO3 for PP4 in the plot), the effects of these PP settings are immediately shown in the lower half of the plot, where the new probability of the CQA conforming to drug substance specifications (depicted as red line) can be seen. 

This real-time prediction combined with the previously mentioned improvements allows for the probability of an OOS event to be calculated ahead of time and enables countermeasures to be taken as necessary. Furthermore, because predictions of scale-dependent process performance characteristics are now also included, the IPM can be used not only as a development tool for setting up an evaluating control strategy, but also as a manufacturing companion to optimize the process in terms of performance and quality. 

## 5. Conclusions

The combined improvements of this IPM represent substantial progress in the development of a bioprocess DA. The original framework’s conceptual advantages were kept while simplifying utilization, and expanding the scope, statistical rigor, applicability, and quality and business objectives. 

As a real-time DA, the IPM allows for simulations during which PP settings can be quickly and seamlessly updated at the moment when new data are observed. Moreover, as further data become available, they may be immediately added via APIs from data sources to refit the model object. This provides the feedback loop both for observed parameter settings and model refitting, crucially enabling the IPM to function as a true DA within a DT concept.

Nonetheless, a substantial part of the improvements relies on the consistent testing of starting material CQAs, which is not universally performed. Thus, to gain benefits, more investment is needed in ensuring as comprehensive a testing plan as possible. While this does not need to be exhaustive, an adequate testing strategy should be built to provide sufficient CQA data at critical junctures to adequately profit from this procedure.

Further development should also be considered here. As this data model increasingly combines large- and small-scale data in the same data matrix, we see particular interest in the investigation of differences in scale behavior, offset, and variances where current scale- down model qualifications are limited. The ease of comparing scales may motivate manufacturing managers to perform runs at the edge of normal operating ranges to gain insight into interaction effects with PPs while avoiding the risk of OOS results. 

Moreover, the IPM technology could be used not only as a tool for control strategy development and deviation management, but also for planning experiments. For example, simulated spiking studies could be used to show which experiments would be needed to identify design space adaptations to decrease the OOS probability in a data-driven manner.

Ultimately, a holistic DA for a simple and robust bioprocess digital twin is eminently feasible and should continue to mature as an essential modeling tool in bioprocess development and manufacturing. 

## Figures and Tables

**Table 1:** Model availability for *Step Yield*.

UO	*Step Yield*
UO1	*Starting UO*
UO2	PP
UO3	PP
UO4	*No model found*
UO5	PP
UO6	PP
UO7	PP
UO8	PP

## Data Availability

The data are not available due their proprietary nature with regards to the industry partner. Certain blinded data is available in the supplementary material.

## Supplementary Materials

The following are available online at: https://www.mdpi.com/article/10.3390/bioengineering9100534/s1. Table S1: overview of identified models based on DoE data; Table S2: step yield data sampled from different campaigns/scales.
