# Supplementary material for: Architectural and Technological Improvements to Integrated Bioprocess Models towards Real-Time Applications
Source: Bioengineering (Basel). 2022 Oct 9;9(10):534. doi: 10.3390/bioengineering9100534 (PMC9598293; doi:10.3390/bioengineering9100534)
Supplement: Supplementary file 1 [file bioengineering-09-00534-s001.zip › bioengineering-1929434-supplementary for conversion.pdf]

Supplemental Data and Plots

Integrated Bioprocess Model: Improvements, Case Study and Real Time Application

Supplementary Models & Data

Architectural & Technological Improvements to Integrated Bioprocess Models towards Real-Time Applications

Table S1: Overview of identified models based on DoE data. AIC was used as a primary identifier of selected model. R^2, Q^2, RMSE, and p-values, as well as residual analysis, were used alongside process expertise to determine acceptance of the model within the IPM. All residual variation is accounted for within the prediction interval of the simulated values within the IPM. (+) indicates positive coefficient and (-) indicates negative coefficient. (cat) indicates a categorical effect, for which there are multiple levels, with either (+) or (-) coefficients from the intercept

Response	Unit Op	Model	R^2	Q^2	RMSE	P	Parameters
Step Yield	UO1	Starting UO (100%)	-	-	-	-	-
	UO2	Linear	0.96	0.92	5.25	<0.0001	(cat) Campaign
	UO3	Linear	0.47	0.38	5.39	<0.0001	(cat) Campaign
	UO4	No model found	-	-	-	-	-
	UO5	Quadratic Interaction	0.72	0.59	8.44	<0.0001	(+) parameter1
							(-) parameter1²
							(+) parameter2
							(+) parameter3
							(+) parameter2*parameter3
	UO6	Quadratic	0.26	0.15	10.98	0.0066	(cat) Campaign
							(+) parameter1
							(+) parameter1²
	UO7	Quadratic	0.17	0.06	5.82	0.0193	(-) parameter1
							(+) parameter1²
	UO8	Quadratic	0.33	0.14	10.63	0.0007	(cat) Campaign
							(-) parameter1
							(+) paramter2
							(-) paramter2²

Supplemental Data and Plots

Integrated Bioprocess Model: Improvements, Case Study and Real Time Application

Table S2: Step Yield Data sampled from different campaigns/scales. Missing data were discussed with process experts and confirmed before model fitting. DoE Data not included in order to compare data against expected manufacturing data.

Batch	Step Yield							
	UO1	UO2	UO3	UO4	UO5	UO6	UO7	UO8
C1 Batch1	100	90.7	87.1	95.7	87.5			
C1 Batch2	100	93.4	91.0	98.7	78.0	52.1	50.5	93.4
C1 Batch3	100	103.8	87.2	91.8	82.8	89.3	61.4	90.4
C1 Batch4	100	98.0	88.0	90.5	93.1	93.8	52.7	83.1
C1 Batch5	100	96.2	81.2	93.5	90.7	66.9	62.4	80.4
C1 Batch6	100	93.4	85.0	97.2	89.8	92.0	53.9	81.9
C1 Batch7	100	90.6	83.6	96.3	91.2	86.2	52.6	89.8
C1 Batch8	100	96.7	78.6	97.7	84.0	88.4	56.5	86.6
C1 Batch9	100	88.0	92.5	91.1	87.1	89.0	55.2	86.6
C1 Batch10	100	98.2	86.4	95.3	74.5	76.0	56.0	83.4
C1 Batch11	100	86.0	94.1	94.5	73.1	79.7	53.6	81.2
C1 Batch12	100	90.9	76.0	96.1	69.6	74.3	59.2	91.5
C2 Batch1	100	106.6	83.7	102.1	82.9	96.4	58.7	73.4
C2 Batch2	100	108.8	81.1	99.9	62.8	83.0	58.6	77.1
C2 Batch3	100	100.3	78.8	104.6	76.3	119.4	42.7	82.8
C2 Batch4	100	98.2	74.5	112.2	69.8	90.7	57.0	81.4
C2 Batch5	100	90.4	98.9	79.2	88.9			82.2
C2 Batch6	100	108.5	76.0	83.8	81.9	93.9	39.2	72.6
C3 Batch1	100		88.8	100.6	79.0	89.9	64.4	80.2
C3 Batch2	100		94.7	92.6	83.5	88.5	59.0	90.3
C3 Batch3	100		90.8	97.2	83.1	82.6	50.3	61.9
C3 Batch4	100		85.4	99.2	83.2	89.7	46.6	105.6
C3 Batch5	100		89.0	97.6	116.0	60.7	45.8	
C4 Batch1	100	80.1	93.2	100.2	81.0	96.9	63.1	75.4
C4 Batch2	100	100.1	99.4	90.3	81.4	88.8	56.0	86.5
C4 Batch3	100	95.1	99.7	89.4	84.1	95.3	55.9	75.7
C4 Batch4	100	91.3	101.2	87.5	91.6	96.5	59.3	53.9
C4 Batch5	100	89.5	93.6	100.4	87.3	102.1	55.0	83.9
C4 Batch6	100	92.5	99.5	106.5	79.3	100.7	50.9	72.8
C4 Batch7	100	93.5	101.3	91.3	91.1	94.2	61.4	74.3
C4 Batch8	100	92.0	93.7	102.6	81.3	78.6	65.2	68.9
C4 Batch9	100	97.4	93.7	100.5	81.0	102.9	54.9	81.6
C4 Batch10	100	94.0	104.3	91.2	87.6	99.8	56.9	78.2
C4 Batch11	100	95.6	88.8	94.9	89.5	93.7	53.1	85.0
C4 Batch12	100	101.1	97.5	90.9	83.6	92.9	54.8	98.9
C4 Batch13	100	94.4	95.6	93.3	81.2	101.9	55.8	78.8
C4 Batch14	100	94.9	91.9	111.3	73.4	86.4	61.6	72.8
C4 Batch15	100	99.4	94.9	101.1	73.8	75.9	66.1	89.0
